# Analysis of eigenvalue condition numbers for a class of randomized numerical methods for singular matrix pencils

**DOI:** 10.1007/s10543-024-01033-w

**Published:** 2024-07-15

**Authors:** Daniel Kressner, Bor Plestenjak

**Affiliations:** 1 Institute of Mathematics, EPFL, 1015 Lausanne, Switzerland; 2 Faculty of Mathematics and Physics, University of Ljubljana, and Institute of Mathematics, Physics and Mechanics, Jadranska 19, 1000 Ljubljana, Slovenia

**Keywords:** Singular pencil, Singular generalized eigenvalue problem, Eigenvalue condition number, Randomized numerical method, Random matrices, 65F15, 15A18, 15A22, 15A21, 47A55, 68W20, 15B52

## Abstract

The numerical solution of the generalized eigenvalue problem for a singular matrix pencil is challenging due to the discontinuity of its eigenvalues. Classically, such problems are addressed by first extracting the regular part through the staircase form and then applying a standard solver, such as the QZ algorithm, to that regular part. Recently, several novel approaches have been proposed to transform the singular pencil into a regular pencil by relatively simple randomized modifications. In this work, we analyze three such methods by Hochstenbach, Mehl, and Plestenjak that modify, project, or augment the pencil using random matrices. All three methods rely on the normal rank and do not alter the finite eigenvalues of the original pencil. We show that the eigenvalue condition numbers of the transformed pencils are unlikely to be much larger than the $\delta$-weak eigenvalue condition numbers, introduced by Lotz and Noferini, of the original pencil. This not only indicates favorable numerical stability but also reconfirms that these condition numbers are a reliable criterion for detecting simple finite eigenvalues. We also provide evidence that, from a numerical stability perspective, the use of complex instead of real random matrices is preferable even for real singular matrix pencils and real eigenvalues. As a side result, we provide sharp left tail bounds for a product of two independent random variables distributed with the generalized beta distribution of the first kind or Kumaraswamy distribution.

## Introduction

The purpose of this work is to study three recent numerical methods, introduced in [12, 13], for computing finite eigenvalues of a square singular matrix pencil $A-\lambda B$, that is, $A,B\in \mathbb C^{n\times n}$ and $\det (A-\lambda B)\equiv 0$. We say that $\lambda _0\in \mathbb C$ is an *eigenvalue* of $A-\lambda B$ if ${\text {rank}}(A-\lambda _0 B) < {\text {nrank}}(A,B)$, where

$$\begin{aligned} \textrm{nrank}(A,B):= \max _{\zeta \in \mathbb C} {\text {rank}}(A-\zeta B)<n \end{aligned}$$

is *the normal rank* of the pencil. Similarly, if ${\text {rank}}(B) < {\text {nrank}}(A,B)$ then we say that $A-\lambda B$ has infinite eigenvalue(s).

A major difficulty when working with a singular pencil numerically is the discontinuity of its eigenvalues, that is, the existence of (arbitrarily small) perturbations of *A*, *B* that completely destroy the eigenvalue accuracy. To circumvent this phenomenon, it is common to first extract the regular part from the staircase form of the pencil [28] before applying the QZ algorithm [15, 22] to compute the eigenvalues. Notably, the popular software GUPTRI [2, 3] is based on this approach. Numerically, the computation of the staircase form requires several rank decisions and these decisions tend to become increasingly difficult as the algorithm proceeds, which can ultimately lead to a failure of correctly identifying and extracting the regular part [7, 23].

Despite the discontinuity of the eigenvalues mentioned above, Wilkinson [30] observed that the QZ algorithm directly applied to the original singular pencil usually returns the eigenvalues of the regular part with reasonable accuracy. De Terán, Dopico, and Moro [1] explained this phenomenon by developing a perturbation theory for singular pencils, implying that the set of perturbation directions causing discontinuous eigenvalue changes has measure zero. Later on, Lotz and Noferini [18] turned this theory into quantitative statements, by measuring the set of perturbations leading to large eigenvalue changes and defining the notion of weak eigenvalue condition number for singular pencils.

It is important to note that Wilkinson’s observation does not immediately lead to a practical algorithm, because the (approximate) eigenvalues returned by the QZ algorithm are mixed with the spurious eigenvalues originating from the (perturbed) singular part and it is a nontrivial task to distinguish these two sets. During the last few years, several methods have been proposed to circumvent this difficulty.

Inspired by the findings in [1], Hochstenbach, Mehl, and Plestenjak [12] proposed to introduce a modification of the form

1 $$\begin{aligned} {\widetilde{A}}-\lambda {\widetilde{B}}:= A-\lambda B+\tau \, (UD_AV^*-\lambda \, UD_BV^*) \end{aligned}$$

for matrices $D_A,D_B\in \mathbb C^{k\times k}$ with $k:= n-{\text {nrank}}(A,B)$, random matrices $U,V\in \mathbb C^{n\times k}$, and a scalar $\tau \not = 0$. Generically, ${\widetilde{A}}-\lambda {\widetilde{B}}$ is a regular pencil and the regular part of $A-\lambda B$ is exactly preserved, i.e., if $\lambda _i$ is a finite eigenvalue of $A-\lambda B$ then $\lambda _i$ is an eigenvalue of ${\widetilde{A}}-\lambda {\widetilde{B}}$ with the same partial multiplicities. More specifically, $\lambda _i$ is an eigenvalue of $A-\lambda B$ if and only if $\lambda _i$ is an eigenvalue of ${\widetilde{A}}-\lambda {\widetilde{B}}$ such that its right/left eigenvectors *x*, *y* satisfy $V^*x=0$ and $U^*y=0$. The latter property is used to extract the eigenvalues of $A-\lambda B$ from the computed eigenvalues of ${\widetilde{A}}-\lambda {\widetilde{B}}$.

In [13], two different variations of the approach from [12] described above are proposed. Instead of adding a modification, the pencil is projected to the generically regular pencil $U_\perp ^*AV_\perp -\lambda U_\perp ^*BV_\perp$ for random matrices $U_\perp ,V_\perp \in \mathbb C^{n\times (n-k)}$, and the eigenvalues of $A-\lambda B$ are extracted from the computed eigenvalues of the smaller pencil. The third method analyzed in this work consists of computing the eigenvalues of $A-\lambda B$ from the augmented generically regular pencil

$$\begin{aligned} \left[ \begin{array}{cc} A &{} UT_A \\ S_AV^* &{} 0 \end{array} \right] - \lambda \, \left[ \begin{array}{cc} B &{} UT_B \\ S_BV^* &{} 0 \end{array} \right] , \end{aligned}$$

where $S_A,S_B,T_A,T_B\in \mathbb C^{k\times k}$ and $U,V\in \mathbb C^{n\times k}$ are random matrices. For both variants, it holds generically that the regular part of $A-\lambda B$ is fully preserved in exact arithmetic. Note that roundoff error affects this eigenvalue preservation property when forming the modified pencils (1) and $U_\perp ^*AV_\perp -\lambda U_\perp ^*BV_\perp$ in finite precision.

One goal of this work is to show that the modifications introduced by the three methods above are numerically safe. More specifically, we show that, with high probability, the eigenvalue condition numbers of the modified pencils are not much larger than the weak eigenvalue condition numbers of the original pencil. In particular, these methods can be expected to return good accuracy for well-conditioned eigenvalues of $A-\lambda B$ in the presence of roundoff error. Another implication of our result is that the eigenvalue condition numbers of the modified pencils represent a reliable complementary criterion for identifying reasonably well-conditioned finite eigenvalues in any of the algorithms from [12, 13].

*Related work* Closer to the analyses in [1, 18], it was recently suggested in [17] to perturb the full pencil: $A+\tau E - \lambda (B+\tau F)$, where $E,F\in \mathbb C^{n\times n}$ are random Gaussian matrices and $\tau >0$ is small but well above the level of machine precision. Unlike for the three methods mentioned above, the regular part of $A-\lambda B$ is not preserved by this perturbation. On the other hand, the direct connection to [18] allows to facilitate their analysis and use the computed eigenvalue condition numbers of the perturbed pencil as a criterion to identify finite eigenvalues of the original pencil. In [17, P. 2], it was stated that a similar analysis would be more difficult for the method from [12] because of the structure imposed on the random perturbation in (1). In this work, we will address this question and carry over the analysis from [17, 18] to the three methods above. In particular, our analysis confirms that the computed eigenvalue condition numbers can be used as a reliable indicator for such algorithms as well.

*Outline* The structure of the paper is as follows. In Sect. 2 we review basic concepts for singular pencils as well as $\delta$-weak condition numbers. In Sect. 3 we present the three randomized numerical methods that we analyze in Sect. 4, where we also obtain the new left tail bounds. This is followed by numerical examples in Sect. 5. In the appendix we provide results obtained with symbolic computation that verify the results from latter sections.

## Preliminaries

### Reducing subspaces and eigenvectors

In order to define eigenvectors of a singular pencil according to [1, 18], we first introduce the Kronecker canonical form (KCF) and the notion of minimal reducing subspaces, see, e.g., [9, 29].

#### Theorem 1

(Kronecker canonical form) Let $A,B\in \mathbb C^{n\times n}$. Then there exist nonsingular matrices $P, Q\in \mathbb C^{n\times n}$ such that

2 $$\begin{aligned} P\,(A-\lambda B)\,Q=\left[ \begin{array}{cc} R(\lambda )&{}0\\ 0 &{} S(\lambda )\end{array}\right] , \qquad R(\lambda )=\left[ \begin{array}{cc}J-\lambda I_r&{}0\\ 0&{}I_s-\lambda N\end{array}\right] , \end{aligned}$$

where *J* and *N* are in Jordan canonical form with *N* nilpotent. Furthermore,

$$\begin{aligned} S(\lambda )= \textrm{diag}\big (L_{m_1}(\lambda ), \dots , L_{m_k}(\lambda ), \ L_{n_{1}}(\lambda )^T, \dots , L_{n_{k}}(\lambda )^T\big ), \end{aligned}$$

where $L_{j}(\lambda )=[0 \ \, I_{j}]-\lambda \, [I_{j} \ \, 0]$ is of size $j\times (j+1)$, and $m_i, n_i\ge 0$ for $i=1,\dots ,k$, where $k=n-\textrm{nrank}(A,B)$.

The pencil $R(\lambda )$ in (2) is called *the regular part* of $A-\lambda B$ and contains the eigenvalues of $A-\lambda B$, where *the Jordan part* $J-\lambda I_r$ contains *the Jordan blocks* of the finite eigenvalues of $A-\lambda B$. If $\lambda _0$ is a finite eigenvalue of $A-\lambda B$, then its *partial multiplicities* are the sizes of the Jordan blocks associated with $\lambda _0$. A finite eigenvalue is called simple if it is a simple root of $\det R(\lambda )$. The pencil $S(\lambda )$ is called *the singular part* of $A-\lambda B$ and contains *right singular blocks* $L_{m_1}(\lambda ),\dots ,L_{m_k}(\lambda )$ and *left singular blocks* $L_{n_{1}}(\lambda )^T,\dots ,L_{n_{k}}(\lambda )^T$, where $m_1,\dots ,m_k$ and $n_{1},\dots ,n_{k}$ are called the *right* and *left minimal indices* of the pencil, respectively.

We say that a subspace ${{\mathcal {M}}}$ is a *reducing subspace* [29] for the pencil $A-\lambda B$ if $\textrm{dim}(A{{\mathcal {M}}} + B{{\mathcal {M}}}) = \textrm{dim}({{\mathcal {M}}}) - k$, where $k=n-\textrm{nrank}(A,B)$ counts the number of right singular blocks. *The minimal reducing subspace* ${{\mathcal {M}}}_{\textrm{RS}}(A,B)$ is the intersection of all reducing subspaces and is spanned by the columns of *Q* corresponding to the blocks $L_{m_1}(\lambda ),\ldots ,L_{m_k}(\lambda )$. Analogously, ${{\mathcal {L}}}$ is a *left reducing subspace* for the pencil $A-\lambda B$ if $\textrm{dim}(A^*{{\mathcal {L}}}+B^*{{\mathcal {L}}})=\textrm{dim}({{\mathcal {L}}}) - k$ and *the minimal left reducing subspace* ${{\mathcal {L}}}_{\textrm{RS}}(A,B)$ is the intersection of all left reducing subspaces.

For an eigenvalue $\lambda _0\in \mathbb C$ of $A-\lambda B$, a nonzero vector $x\in \mathbb C^n$ is called a *right eigenvector* if $(A-\lambda _0 B)x=0$ and $x\not \in {{\mathcal {M}}}_{\textrm{RS}}(A,B)$. A nonzero vector $y\in \mathbb C^n$ such that $y^*(A-\lambda _0 B)=0$ and $y\not \in {{\mathcal {L}}}_{\textrm{RS}}(A,B)$ is called a *left eigenvector*. This agrees with the definition of eigenvectors from [5, 18]. Compared to a regular pencil, the eigenvectors of singular pencils have a much larger degree of non-uniqueness, due to components from the minimal reducing subspaces.

### Eigenvalue perturbation theory

Suppose that we perturb an $n\times n$ matrix pencil $A-\lambda B$ into

3 $$\begin{aligned} {\widetilde{A}}-\lambda {\widetilde{B}}:=A+\epsilon E-\lambda (B+\epsilon F), \end{aligned}$$

where $\epsilon >0$. We define $\Vert (E,F)\Vert :=(\Vert E\Vert _F^2+\Vert F\Vert _F^2)^{1/2}$, where $\Vert \cdot \Vert _F$ denotes the Frobenius norm of a matrix. When $\Vert (E,F)\Vert =1$ we can identify the pencil $E-\lambda F$ with a point on the unit sphere in $\mathbb C^{2n^2}$ and think of (*E*, *F*) as a direction of the perturbation (3).

Before addressing the singular case, let us first recall the classical eigenvalue perturbation theory [27] for a *regular* pencil $A-\lambda B$. Consider a simple finite eigenvalue $\lambda _0\in \mathbb C$ of $A-\lambda B$ with normalized right/left eigenvectors *x*, *y*. For $\Vert (E,F)\Vert =1$ and sufficiently small $\epsilon >0$ there exists an eigenvalue $\lambda _0(\epsilon )$ of the perturbed pencil (3) satisfying the perturbation expansion

4 $$\begin{aligned} |\lambda _0(\epsilon )-\lambda _0|= \frac{|y^*(E-\lambda _0 F)x|}{|y^*Bx|}\epsilon +{{\mathcal {O}}}(\epsilon ^2) \le \frac{(1+|\lambda _0|^2)^{1/2}}{|y^*Bx|}\epsilon +{{\mathcal {O}}}(\epsilon ^2) \end{aligned}$$

as $\epsilon \rightarrow 0$. Note that the inequality becomes an equality for the direction $E=(1+|\lambda _0|^2)^{-1/2}yx^*$, $F=-{\overline{\lambda }}_0E$. In turn, the *absolute condition number* of $\lambda _0$, defined as

5 $$\begin{aligned} \kappa (\lambda _0)=\lim _{\epsilon \rightarrow 0}\sup _{\Vert (E,F)\Vert \le 1} \frac{1}{\epsilon }|\lambda _0(\epsilon )-\lambda _0|, \end{aligned}$$

satisfies

6 $$\begin{aligned} \kappa (\lambda _0)=1 / \gamma (\lambda _0),\ \textrm{where}\ \gamma (\lambda _0)=|y^*Bx|(1+|\lambda _0|^2)^{-1/2}; \end{aligned}$$

see, e.g., [8, Lemma 3.1 and Eq. (3.3)].

For a singular pencil, the definition (5) always leads to an infinite condition number because of the discontinuity of eigenvalues. To address this, we first recall the eigenvalue expansion by De Terán, Dopico, and Moro [1, Corollary 2].

#### Theorem 2

Let $\lambda _0$ be a finite simple eigenvalue of an $n\times n$ pencil $A-\lambda B$ of normal rank $n-k$, $k\ge 1$. Let $X=[X_1\ x]$, $Y=[Y_1\ y]$ be $n\times (k+1)$ matrices with orthonormal columns such that: $X_1$ is a basis for $\textrm{ker}(A-\lambda _0 B)\cap {{\mathcal {M}}}_{\textrm{RS}}(A,B)$, *X* is a basis for $\textrm{ker}(A-\lambda _0 B)$, $Y_1$ is a basis for $\textrm{ker}((A-\lambda _0 B)^*)\cap {{\mathcal {L}}}_{\textrm{RS}}(A,B)$, and *Y* is a basis for $\textrm{ker}((A-\lambda _0 B)^*)$. If $E-\lambda F$ is such that $\det (Y_1^*(E-\lambda _0 F)X_1)\ne 0$, then, for sufficiently small $\epsilon >0$, there exists an eigenvalue $\lambda _0(\epsilon )$ of the perturbed pencil (3) such that

7 $$\begin{aligned} \lambda _0(\epsilon )=\lambda _0 - \frac{\det (Y^*(E-\lambda _0 F)X)}{y^*Bx\cdot \det (Y_1^*(E-\lambda _0 F)X_1)}\epsilon +{{\mathcal {O}}}(\epsilon ^2). \end{aligned}$$

The expansion (7) allows one to define *the directional sensitivity* for perturbations $E-\lambda F$, $\Vert (E,F)\Vert =1$, satisfying the condition of Theorem 2:

8 $$\begin{aligned} \sigma _{E,F}(\lambda _0)=\left| \frac{\det (Y^*(E-\lambda _0 F)X)}{y^*Bx\cdot \det (Y_1^*(E-\lambda _0 F)X_1)}\right| ; \end{aligned}$$

see [18, Definition 2.4 and Corollary 3.3]. We can now generalize the definition of $\gamma (\lambda _0)$ in (6) to singular pencils by using the right/left eigenvectors *x*, *y* from Theorem 2. Because of the appearance of the factor $y^*Bx$ in the denominator of (8), we expect that the quantity $1/\gamma (\lambda _0)$ continues to play a crucial role in determining the sensitivity of an eigenvalue. However, it is important to note that *x*, *y* are particular choices of eigenvectors made in Theorem 2: the right eigenvector *x* is orthogonal to the right minimal reducing subspace ${{\mathcal {M}}}_{\textrm{RS}}$ and the left eigenvector *y* is orthogonal to ${{\mathcal {L}}}_{\textrm{MR}}$. Under these constraints, the eigenvectors *x* and *y* of the simple eigenvalue $\lambda _0$ become uniquely determined up to multiplication by unit complex numbers. It follows from $Y_1^* B X_1 = 0$, $Y_1^*Bx=0$ and $y^*BX_1=0$ that this particular choice maximizes $|y^*B x|$, i.e., for all vectors *z* and *w* such that $\Vert z\Vert _2=\Vert w\Vert _2=1$, $(A-\lambda _0 B)z=0$, and $w^*(A-\lambda _0 B)=0$, it holds that

9 $$\begin{aligned} |w^*Bz|(1+|\lambda _0|^2)^{-1/2} \le \gamma (\lambda _0). \end{aligned}$$

#### Remark 3

If *A*, *B* are real matrices and $\lambda _0$ is a real simple eigenvalue, then the matrices $X,X_1,Y,Y_1$ and vectors *x*, *y* in Theorem 2 can be chosen to be real as well.

Clearly, the perturbations for which the quantity $\det (Y_1^*(E-\lambda _0 F)X_1)$ in the denominator of (7) vanishes form a set of measure zero in the unit sphere in $\mathbb C^{2n^2}$. In other words, this event has zero probability if we draw (*E*, *F*) uniformly at random from the the unit sphere in $\mathbb C^{2n^2}$, which we will denote by $(E,F)\sim {{\mathcal {U}}}(2n^2)$. The $\delta$-*weak condition number* introduced by Lotz and Noferini [18, Definition 2.5] offers a more refined picture by measuring the tightest upper bound *t* such that the directional sensitivity (8) stays below *t* with probability at least $1-\delta$.

#### Definition 1

Let $\lambda _0\in \mathbb C$ be a finite simple eigenvalue of a singular pencil $A-\lambda B$. The $\delta$-*weak condition number* of $\lambda _0$ is defined as

$$\begin{aligned} \kappa _w(\lambda _0;\delta )=\inf \big \{t\in \mathbb R:\ \mathbb {P}(\sigma _{E,F}(\lambda _0)<t)\ge 1-\delta \big \},\quad (E,F)\sim {{\mathcal {U}}}(2n^2). \end{aligned}$$

If $\lambda _0$ is a finite simple eigenvalue of a regular pencil $A-\lambda B$, it follows from (3) and (6) that $\sigma _{E,F}(\lambda _0)=|y^*(E-\lambda _0 F)x|/|y^*Bx|\le \kappa (\lambda _0)=1/\gamma (\lambda _0)$ for all $\Vert (E,F)\Vert =1$. Therefore, if we apply Definition 1 to a regular pencil, it follows from (5) that $\kappa _w(\lambda _0;\delta )$ converges to $\kappa (\lambda _0) = 1/\gamma (\lambda _0)$ monotonically from below as $\delta \downarrow 0$. The following result from [18, Theorem 5.1] and [17, Theorem 3.1] suggests to use $1/\gamma (\lambda _0)$ as a proxy for eigenvalue sensitivity in the singular case as well.

#### Theorem 4

Let $\lambda _0\in \mathbb C$ be a finite simple eigenvalue of an $n\times n$ singular pencil $A-\lambda B$ of normal rank $n-k$. Then for $\delta \le k / (2n^2)$ it holds that

$$\begin{aligned} \frac{1}{\sqrt{\delta 2n^2}} \cdot \frac{1}{\gamma (\lambda _0)} \le \kappa _w(\lambda _0;\delta )\le \frac{\sqrt{k}}{\sqrt{\delta 2n^2}} \cdot \frac{1}{\gamma (\lambda _0)}, \end{aligned}$$

where $\gamma (\lambda _0)$ is defined as in (6) with the right/left eigenvectors *x*, *y* from Theorem 2.

The algorithms for the singular generalized eigenvalue problem presented in the next section use $\gamma (\lambda _0)$ to identify (finite) eigenvalues numerically.

We finish this section by remarking that the absolute condition number (5) is consistent with the definitions in [17, 18]. Following, e.g., [10] one could also consider a notion of relative condition number that imposes $\Vert E\Vert _F\le \Vert A\Vert _F$ and $\Vert F\Vert _F\le \Vert B\Vert _F$ instead of $\Vert (E,F)\Vert \le 1$ on the perturbation direction in (3). In effect, both the standard and weak (absolute) condition numbers get multiplied by the factor $(\Vert A\Vert _F^2+\Vert B\Vert _F^2)^{1/2}$. Under reasonable choices of the parameters involved, this additional factor does not differ significantly for the modified pencils. In particular, our results presented for absolute condition numbers easily extend to relative condition numbers.

## Randomized numerical methods based on the normal rank

In this section, we describe in some detail the three numerical methods from [12, 13] for computing the finite eigenvalues of an $n\times n$ singular pencil $A-\lambda B$ with $\textrm{nrank}(A,B)=n-k$ for $k \ge 1$. All three methods require knowledge about the exact normal rank in order to leave the regular part intact. If this quantity is not known a priori, it can be determined from $\textrm{rank}(A-\xi _i B)$ for a small number of randomly chosen $\xi _i\in \mathbb C$.^1^

### Rank-completing modification

We first consider the rank-completing method from [12], where a random pencil of normal rank *k* is added to yield a (generically regular) matrix pencil

10 $$\begin{aligned} {\widetilde{A}}-\lambda {\widetilde{B}}:= A-\lambda B+\tau \, (UD_AV^*-\lambda \, UD_BV^*), \end{aligned}$$

where $D_A,D_B\in \mathbb C^{k\times k}$ are diagonal matrices such that $D_A-\lambda D_B$ is regular, $U,V\in \mathbb C^{n\times k}$ are matrices of rank *k*, and $\tau \in \mathbb C$ is nonzero. Note that *k* is the smallest normal rank for such a modification to turn a singular into a regular pencil. The following result [12, Summary 4.7], see also [13, Remark 3.5], characterizes the dependence of eigenvalues and eigenvectors of the modified pencil (10) on $\tau$, $D_A$, $D_B$, *U*, and $V^*$.

#### Summary 5

Let $A-\lambda B$ be an $n\times n$ singular pencil of normal rank $n-k$ with left minimal indices $n_1,\dots ,n_k$ and right minimal indices $m_1,\dots ,m_k$. Let $N = n_1 + \cdots + n_k$ and $M = m_1 + \cdots + m_k$. Then the regular part of $A-\lambda B$ has size $r:=n-N-M-k$ and generically (with respect to the entries of $D_A,D_B,U,V^*$), the modified pencil ${\widetilde{A}}-\lambda {\widetilde{B}}$ defined in (10) is regular and its eigenvalues are classified in the following four disjoint groups:

1. *True eigenvalues*: There are *r* eigenvalues counted with their multiplicities that coincide with the eigenvalues of the original pencil $A-\lambda B$ with the same partial multiplicities. The right/left eigenvectors *x*, *y* of ${\widetilde{A}}-\lambda {\widetilde{B}}$ belonging to these eigenvalues satisfy the orthogonality relations $V^*x=0$ and $U^*y=0$.
2. *Prescribed eigenvalues*: There are *k* eigenvalues such that $V^*x\ne 0$ for all right eigenvectors *x* and $U^*y\ne 0$ for all left eigenvectors *y*. These are the *k* eigenvalues of $D_A-\lambda D_B$.
3. *Random right eigenvalues*: There are *M* eigenvalues, which are all simple and such that $V^*x=0$ for all right eigenvectors *x* and $U^*y\ne 0$ for all left eigenvectors *y*.
4. *Random left eigenvalues*: There are *N* eigenvalues, which are all simple and such that $V^*x\ne 0$ for all right eigenvectors *x* and $U^*y=0$ for all left eigenvectors *y*.

Summary 5 has the following practical consequences. If we compute all eigenvalues $\lambda _i$ of (10), together with the (normalized) right and left eigenvectors $x_i$ and $y_i$ for $i=1,\ldots ,n$, then $\max (\Vert V^*x_i\Vert _2,\Vert U^*y_i\Vert _2)=0$ if and only if $\lambda _i$ is an eigenvalue of $A - \lambda B$. In numerical computations, we can use $\max (\Vert V^*x_i\Vert _2,\Vert U^*y_i\Vert _2)<\delta _1$, where $\delta _1$ is a prescribed threshold, as a criterion to extract the true eigenvalues in the first phase. Note that for a simple finite eigenvalue $x_i$ and $y_i$ are unique (up to multiplication by unit complex numbers) because, *generically*, the *modified* pencil is regular. They correspond to eigenvectors of the original singular pencil satisfying the orthogonality constraints $V^*x_i =0$ and $U^*y_i =0$.

In the second phase, we use the (reciprocal) eigenvalue sensitivities for extracting simple finite eigenvalues, that is, we compute

11 $$\begin{aligned} \gamma _i=|y_{i}^*Bx_i|(1+|\lambda _i|^2)^{-1/2} \end{aligned}$$

and identify $\lambda _i$ as a finite eigenvalue if $\gamma _i>\delta _2$ for a prescribed threshold $\delta _2$. Note that $1/\gamma _i$ is the absolute condition number of $\lambda _i$ as an eigenvalue of the (generically) regular pencil (10); see (6).

For different matrices *U* and *V* in (10) we obtain different eigenvectors $x_i$ and $y_i$ and thus different values of $\gamma _i$ for the same eigenvalue, while the changing of $\tau$, $D_A$ and $D_B$ does not affect the eigenvectors [13, Lemma 3.4]. In Sect. 4 we will analyze these values for random *U* and *V* and compare them to the unique value $\gamma (\lambda _i)$ that appears in the $\delta$-weak condition number of $\lambda _i$ as an eigenvalue of the singular pencil $A-\lambda B$; see Theorem 4.

The considerations above lead to Algorithm 1 from [12]. In theory, the results returned by the algorithm are independent of $\tau \ne 0$. In practice, $|\tau |$ should be neither too small nor too large in order to limit the impact of roundoff error; in [12] it is suggested to scale *A* and *B* so that $\Vert A\Vert _1=\Vert B\Vert _1=1$, where $\Vert A\Vert _1=\max _{1\le j\le n}\sum _{i=1}^n|a_{ij}|$, and take $\tau =10^{-2}$. The quantity $\varepsilon$ stands for the machine precision.
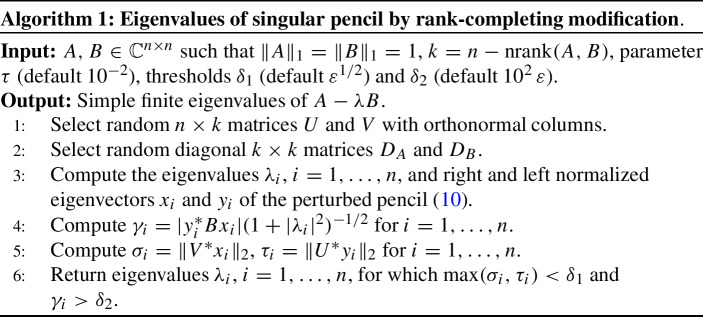


By the theory in [12, 13], the eigenvectors of the modified pencil (10) that correspond to true eigenvalues do not change if we replace *U* and *V* by ${\widetilde{U}}=UR$ and ${\widetilde{V}}=VS$, where *R* and *S* are arbitrary nonsingular $k\times k$ matrices. Thus, choosing *U*, *V* to have orthonormal columns does not violate the genericity assumption in Summary 5.

Note that $\gamma _i$ in line 4 was initially computed in [12] as $\gamma _i=|y_i^*{\widetilde{B}} x_i|$. This was changed to $\gamma _i=|y_i^*{\widetilde{B}} x_i|(1+|\lambda _i|^2)^{-1/2}$ in [13] to be consistent with [17, 18]. Since for true eigenvalues $y_i^*{\widetilde{B}} x_i=y_i^*B x_i$, we use *B* instead of ${\widetilde{B}}$ to simplify the analysis.

We remark that the values $\gamma _i$ from (11) were also used in [17] for computing finite eigenvalues of a singular pencil via unstructured random perturbations. The use of full-rank perturbations comes with two disadvantages: the orthogonality relations for the eigenvectors exploited above are not satisfied and, in contrast to Algorithm 1, the eigenvalues of the perturbed pencil in [17] differ from the exact eigenvalues of the original pencil. The latter leads one to choose $\tau > 0$ very small, but at the same time it needs to stay well above the level of machine precision.

### Normal rank projections

In [13], a variant of Algorithm 1 was proposed that uses random projections to a pencil of smaller size, equal to the normal rank $n-k$. In addition, the method does not require to choose the matrices $D_A$, $D_B$, and the parameter $\tau$.

For $U_\perp , V_\perp \in \mathbb C^{n \times (n-k)}$, we consider the $(n-k)\times (n-k)$ pencil $U_{\perp }^*(A-\lambda B)\,V_{\perp }$. To connect it to the modified pencil (10) used in Algorithm 1, let us assume that the columns of $U_\perp$ and $V_\perp$ span the orthogonal complements of ranges of *U* and *V*, respectively, so that $U_\perp ^* U = 0$, $V_\perp ^* V = 0$. The pencil

$$\begin{aligned} {\widehat{A}}-\lambda {\widehat{B}}&:= [U \ \, U_{\perp }]^*\,(A - \lambda B + \tau (UD_AV^* -\lambda UD_BV^*)\,[V \ \, V_{\perp }] \\&= \left[ \begin{array}{cc} U^*(A-\lambda B)V +\tau (D_A-\lambda D_B) &{} U^*(A-\lambda B)V_{\perp }\\ U_{\perp }^*(A-\lambda B)V &{} U_{\perp }^*(A-\lambda B)V_{\perp } \end{array} \right] \end{aligned}$$

is then equivalent to (10) and we observe the following.

#### Proposition 1

([13, Proposition 4.1 and Theorem 4.2]) Let $A-\lambda B$ be a complex $n\times n$ singular pencil of normal rank $n-k$. Then, under the assumptions of Summary 5, the $(n-k)\times (n-k)$ pencil $A_{22}-\lambda B_{22}:=U_{\perp }^*(A-\lambda B)\,V_{\perp }$ is generically regular and the eigenvalues of $A_{22}-\lambda B_{22}$ are precisely:

- the random eigenvalues of (10) (groups 3 and 4 in Summary 5);
- the true eigenvalues of $A-\lambda B$.

Based on the above results, an algorithm is devised in [13]. Algorithm 2 is a simplified form that matches Algorithm 1 as much as possible.
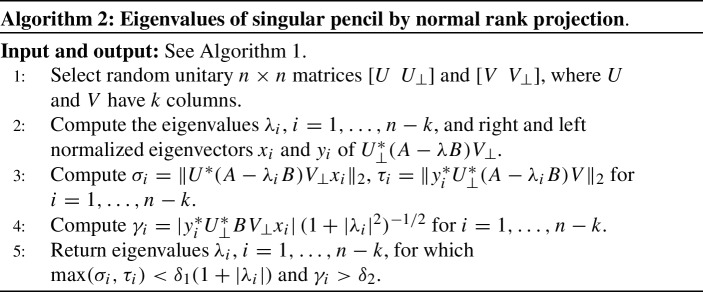


The following corollary shows that the reciprocal eigenvalue condition number $\gamma _i$ computed in line 4 of Algorithm 2 matches the corresponding quantity of Algorithm 1.

#### Corollary 1

Let $\lambda _i\in \mathbb C$ be a simple eigenvalue of a singular pencil $A-\lambda B$. Under the assumptions of Proposition 1, if

- $(\lambda _i,x_i,y_i)$ is an eigentriple of (10) such that $\Vert x_i\Vert _2=1$, $\Vert y_i\Vert _2=1$, $V^*x_i=0$, and $U^*y_i=0$; and
- $(\lambda _i,w_i,z_i)$ is an eigentriple of $A_{22}-\lambda B_{22}$ such that $\Vert w_i\Vert _2=1$, $\Vert z_i\Vert _2=1$, $U^*(A-\lambda _iB)V_\perp w_i=0$, and $z_i^*U_\perp ^*(A-\lambda _iB)V=0$,

then $$|y_i^*Bx_i|=|z_i^*U_\perp ^*BV_\perp w_i|$$.

#### Proof

Since $\lambda _i$ is simple, the vectors $x_i,y_i$ from (a) and $w_i,z_i$ from (b) are uniquely defined up to multiplication by unit complex numbers. If (a) and (b) both hold then it immediately follows that, up to sign changes, $x_i=V_\perp w_i$ and $y_i=U_\perp z_i$. $\square$

### Augmentation

The third method, also presented in [13], uses the $(n+k) \times (n+k)$ augmented (or bordered) matrix pencil

12 $$\begin{aligned} A_a-\lambda B_a:= \left[ \begin{array}{cc} A &{} UT_A \\ S_AV^* &{} 0 \end{array} \right] - \lambda \, \left[ \begin{array}{cc} B &{} UT_B \\ S_BV^* &{} 0 \end{array} \right] , \end{aligned}$$

where $S_A,S_B,T_A$, and $T_B$ are $k\times k$ diagonal matrices and *U*, *V* are $n\times k$ matrices.

#### Proposition 2

([13, Proposition 5.1]) Let $A-\lambda B$ be an $n\times n$ singular pencil of normal rank $n-k$ such that all its eigenvalues are semisimple. Assume that the diagonal $k\times k$ pencils $S_A-\lambda S_B$ and $T_A-\lambda T_B$ are regular and that their 2*k* eigenvalues are pairwise distinct. Furthermore, let $U,V\in \mathbb C^{n\times k}$ have orthonormal columns such that the augmented pencil (12) is regular. Then the pencil (12) has the following eigenvalues:

- 2*k* prescribed eigenvalues, which are precisely the eigenvalues of $S_A-\lambda S_B$ and $T_A-\lambda T_B$;
- the random eigenvalues of (10) (groups 3 and 4 in Summary 5) with the same *U* and *V* and with $D_A=T_AS_A$, $D_B=T_BS_B$;
- the true eigenvalues of $A-\lambda B$.

The algorithm based on the above proposition is given in Algorithm 3.
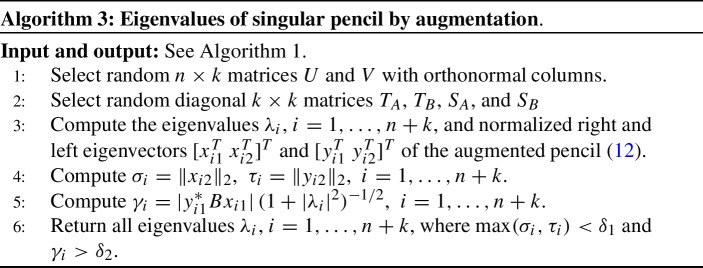


Again, the reciprocal eigenvalue condition number $\gamma _i$ computed in line 5 of Algorithm 3 matches the corresponding quantity of Algorithm 1.

#### Corollary 2

Under the assumptions of Proposition 2, if

- $(\lambda _i,x_i,y_i)$ is an eigentriple of (10) such that $\Vert x_i\Vert _2=1$, $\Vert y_i\Vert _2=1$, $V^*x_i=0$, and $U^*y_i=0$; and
- $(\lambda _i,w_i,z_i)$, where $w_i=[w_{i1}^T\ 0]^T$ and $z_i=[z_{i1}^T\ 0]^T$, such that $w_{i1}\in \mathbb C^n$, $z_{i1}\in \mathbb C^n$, $\Vert w_i\Vert _2=1$, $\Vert z_i\Vert _2=1$, is an eigentriple of the augmented pencil (12),

then $$|y_i^*Bx_i|=|z_i^*B_aw_i|=|z_{i1}^*Bw_{i1}|$$.

#### Proof

If a) and b) are both true then it immediately follows that, up to sign changes, $x_i=w_{i1}$ and $y_i=z_{i1}$.$\square$

Let us remark that Algorithms 1–3, with additional heuristic criteria, can in practice, due to the “positive” effect of roundoff error, successfully compute multiple finite eigenvalues as well; we refer to [13, Sec. 6] for details.

#### Remark 6

In exact arithmetic, a simple finite eigenvalue $\lambda _0$ of a singular pencil $A-\lambda B$ is an exact eigenvalue of a modified *regular* pencil ${\widetilde{A}}-\lambda {\widetilde{B}}$ that we obtain by rank-completion, projection, or augmentation. In finite precision, ${\widetilde{A}}-\lambda {\widetilde{B}}$ is affected by roundoff error. First, the construction of ${\widetilde{A}}-\lambda {\widetilde{B}}$ itself already introduces some error:

13 $$\begin{aligned} ({\widetilde{A}} + \triangle {\widetilde{A}}) - \lambda ({\widetilde{B}} + \triangle {\widetilde{B}} ). \end{aligned}$$

Standard error analysis results [11] imply for all three methods that $\Vert \triangle {\widetilde{A}} \Vert _2 \lesssim {{\textbf{u}}} \Vert A\Vert _2$ and $\Vert \triangle {\widetilde{B}} \Vert _2 \lesssim {{\textbf{u}}} \Vert B\Vert _2$, where ${{\textbf{u}}}$ denotes unit roundoff, provided that the parameters are reasonably chosen. For example, in the augmentation (12), the norms of $T_A, S_A$ should be balanced with the norm of *A* and the norms of $T_B, S_B$ should be balanced with the norm of *B*. The application of the QZ algorithm for computing the eigenvalues of the perturbed pencil (13) introduces further error but, thanks to its backward stability, the additional error is of the same nature. In summary, the computed eigenvalues are the exact eigenvalues of a slightly perturbed *modified* pencil. In turn, we expect the computed approximation for an eigenvalue to be accurate if the (exact) eigenvalue of ${\widetilde{A}}-\lambda {\widetilde{B}}$ is not ill-conditioned. Our analysis will show that if $\lambda _0$ is well-conditioned as an eigenvalue of $A-\lambda B$, then it is – with high probability – also a well-conditioned eigenvalue of ${\widetilde{A}}-\lambda {\widetilde{B}}$. In turn, one can expect good accuracy for well-conditioned eigenvalues of $A-\lambda B$.

Let us stress that it is nontrival to relate (13) back to an error in the original singular pencil $A-\lambda B$. At least, we are not aware of existing backward error analysis results for singular matrix pencils that would allow us to do this.

## Probabilistic analysis

Our goal is to analyze the behavior of the quantities $\gamma _i$ in Algorithms 1–3 and show that they are unlikely to be much below $\gamma (\lambda _i)$. It follows from Corollaries 1 and 2 that it is sufficient to consider Algorithm 1 and the quantity $\gamma _i$ defined in (11).

In the following, we assume that $\lambda _i$ is a simple eigenvalue of $A-\lambda B$ and let $X=[X_1\ x]$ and $Y=[Y_1\ y]$ denote the orthonormal bases for $\textrm{ker}(A-\lambda _i B)$ and $\textrm{ker}((A-\lambda _i B)^*)$ introduced in Theorem 2. We recall from Theorem 4 that the reciprocal of $\gamma (\lambda _i) = |y^* B x| (1+|\lambda _i|^2)^{-1/2}$ critically determines the sensitivity of $\lambda _i$ as an eigenvalue of $A-\lambda B$. The sensitivity of $\lambda _i$ as an eigenvalue of ${\widetilde{A}}-\lambda {\widetilde{B}}$ from (10) is given by $1/\gamma _i$ with $\gamma _i = |y_i^* B x_i| (1+|\lambda _i|^2)^{-1/2}$; see (11). The eigenvectors $x_i, y_i$ are normalized ($\Vert x_i\Vert _2=\Vert y_i\Vert _2=1$) and depend on the choices of *U* and *V* in Algorithm 1. Generically (with respect to $D_A,D_B,U,V^*$), Summary 5.1 yields the relations

14 $$\begin{aligned} x_i=[X_1\ x]\left[ \begin{matrix} a \\ \alpha \end{matrix}\right] \quad {\textrm{and}}\quad y_i=[Y_1\ y]\left[ \begin{matrix} b \\ \beta \end{matrix}\right] ,\quad V^*x_i=0, \quad U^*y_i=0. \end{aligned}$$

If $V^* X_1$ is invertible, the choice of *V* entirely determines $|\alpha |$ because $V^* x_i = 0$ implies $a = -(V^* X_1)^{-1} V^* x \alpha$ and, hence, $\Vert x_i\Vert _2 = 1$ implies

$$\begin{aligned}|\alpha | = 1/\sqrt{1 + \Vert (V^* X_1)^{-1} V^* x\Vert _2^2}.\end{aligned}$$

Analogously, the choice of *U* determines $|\beta |$ if $U^* Y_1$ is invertible.

Since $Y^*BX_1=0$ and $Y_1^*BX=0$, we get $y_i^*Bx_i = \alpha {{\overline{\beta }}} y^*Bx$ and thus

$$\begin{aligned} \gamma _i=|\alpha | |\beta | \gamma (\lambda _i). \end{aligned}$$

The relation $0\le |\alpha |,|\beta |\le 1$ immediately gives $\gamma _i\le \gamma (\lambda _i)$, in line with (9). A small value of $|\alpha ||\beta |$ means an increased eigenvalue sensitivity for ${\widetilde{A}}-\lambda {\widetilde{B}}$, potentially causing Algorithm 1 to yield unnecessarily inaccurate results. In the following, we will show that this is unlikely when random matrices *U*, *V* are used in Algorithm 1. This also implies that the (reciprocal) condition numbers computed in Algorithm 1 can be used with high probability to correctly identify finite simple eigenvalues.

### Preliminary results

Let ${{\mathcal {N}}}^1(\mu ,\sigma ^2)$ denote the normal distribution with mean $\mu$ and variance $\sigma ^2$. In particular, $x\sim {{\mathcal {N}}}^1(0,1)$ is a standard (real) normal random variable. We write $z\sim {{\mathcal {N}}}^2(0,1)$ if $z=x+\textrm{i} y$ is a standard complex normal variable, that is, $x,y\sim {{\mathcal {N}}}^1(0,\frac{1}{2})$ are independent. In the following, we will analyze real matrices (${\mathbb {F}}=\mathbb R$) and complex matrices (${\mathbb {F}}=\mathbb C$) simultaneously. For this purpose, we set $\phi =1$ for ${\mathbb {F}}=\mathbb R$ and $\phi =2$ for ${\mathbb {F}}=\mathbb C$.

The matrices *U* and *V* from Algorithm 1 belong to *the Stiefel manifold*

$$\begin{aligned}\mathbb V_k^n({\mathbb {F}})=\{Q\in {\mathbb {F}}^{n\times k}:\ Q^*Q=I\}.\end{aligned}$$

We will choose them randomly (and independently) from the uniform distribution on $\mathbb V_k^n({\mathbb {F}})$. A common way to compute such a matrix is to perform the QR decomposition of an $n\times k$ *Gaussian random matrix* *M*, that is, the entries of *M* are i.i.d. real or complex standard normal variables, see e.g., [21, 26]. That this indeed yields the uniform distribution follows from the following variant of the well-known Bartlett decomposition theorem ([24, Theorem 3.2.14], [6, Proposition 7.2]); see also [18, Proposition 4.5].

#### Theorem 7

For ${\mathbb {F}}\in \{\mathbb R,\mathbb C\}$, let $M\in {\mathbb {F}}^{n\times k}$, $n \ge k$, be a Gaussian random matrix. Consider the QR decomposition $M=QR$, where $Q\in \mathbb V_k^n({\mathbb {F}})$ and $R\in {\mathbb {F}}^{k\times k}$ is upper triangular with non-negative diagonal entries. Then

- the entries of *Q* and the entries of the upper triangular part of *R* are all independent random variables;
- *Q* is distributed uniformly over $\mathbb V_k^n({\mathbb {F}})$;
- $$r_{ij}\sim {{\mathcal {N}}}^\phi (0,1)$$ for $$1\le i< j\le k$$;
- $$\phi r_{jj}^2\sim \chi ^2(\phi (n-j+1))$$ for $$j=1,\ldots ,k$$;

where $\chi ^2(\ell )$ denotes the chi-squared distribution with $\ell$ degrees of freedom.

Part (b) of Theorem 7 implies that each column of *Q* is distributed uniformly over the unit sphere in ${\mathbb {F}}^n$. Note that $x = z / \Vert z\Vert _2$, for a Gaussian random vector $z \in {\mathbb {F}}^n$, has the same distribution. The following result provides the distribution of the entries of *x*; this result can be found for ${\mathbb {F}}=\mathbb R$ in [4].

#### Lemma 1

Consider a random vector *x* distributed uniformly over the unit sphere in ${\mathbb {F}}^n$ for $n\ge 2$. Then the entries of *x* are i.i.d. with

$$\begin{aligned} |x_i|^2\sim {\textrm{Beta}}\left( \frac{\phi }{2},\frac{\phi (n-1)}{2} \right) , \quad i=1,\ldots ,n, \end{aligned}$$

where ${\textrm{Beta}}$ denotes the beta distribution.

#### Proof

By Theorem 7, the entries of *x* are independent. Without loss of generality, let $i = 1$. Using that $x=z/\Vert z\Vert _2$ for a Gaussian random vector *z* and setting $$w = \left[ \begin{matrix}z_2&\ldots&z_n\end{matrix}\right] $$, it follows that $|x_1|^2=\frac{|z_1|^2}{|z_1|^2+\Vert w\Vert _2^2}$, where $z_1$, *w* are independent and $\phi |z_1|^2\sim \chi ^2(\phi )$, $\phi \Vert w\Vert _2^2\sim \chi ^2(\phi (n-1))$. This implies the claimed result; see, e.g., [6, p. 320]. $\square$

Our analysis will connect $\alpha$ and $\beta$ from (14) to the nullspaces of $k\times (k+1)$ standard Gaussian matrices, which are characterized by the following result.

#### Lemma 2

For a Gaussian random matrix $\varOmega \in {\mathbb {F}}^{k\times (k+1)}$ with $k \ge 2$, let *x* be a vector in the nullspace of $\varOmega$ such that $\Vert x\Vert _2 = 1$. Then, with probability one, $|x_i|$ is uniquely determined and satisfies

15 $$\begin{aligned} |x_i|^2 \sim {\textrm{Beta}}\left( \frac{\phi }{2},\frac{\phi k}{2} \right) . \end{aligned}$$

#### Proof

We assume that $\varOmega$ has rank *k*, which holds with probability 1. For a Gaussian random vector $\omega \in {\mathbb {F}}^{k+1}$ independent of $\varOmega ^*$, consider the QR decomposition $[\varOmega ^*, \omega ] = QR$. Letting *x* denote the last column of *Q*, it follows that *x* is orthogonal to the columns of $\varOmega ^*$ or, in other words, *x* is in the nullspace of $\varOmega$. From Theorem 7, it follows that *x* is distributed uniformly over the unit sphere in ${\mathbb {F}}^{k+1}$. The distribution (15) then follows from Lemma 1. Finally, note that $|x_i|^2$ is uniquely determined because the nullspace of $\varOmega$ has dimension 1. $\square$

### Statistics of $|\alpha |, |\beta |$

The results above readily yield the distribution of $|\alpha |^2$ and $|\beta |^2$.

#### Proposition 3

For ${\mathbb {F}} \in \{\mathbb R, \mathbb C\}$, let *U*, *V* be $n\times k$ independent random matrices from the uniform distribution on the Stiefel manifold $\mathbb V_k^n({\mathbb {F}})$. Consider a finite simple eigenvalue $\lambda _i \in {\mathbb {F}}$ of a singular pencil $A-\lambda B$ with $A,B\in {{\mathbb {F}}}^{n\times n}$. Let $x_i$ and $y_i$ be the right and left normalized eigenvectors of the perturbed regular pencil (10) for the eigenvalue $\lambda _i$ and let $\alpha , \beta$ be defined as in (14). Then $|\alpha |$ and $|\beta |$ are independent random variables and

$$\begin{aligned} |\alpha |^2, |\beta |^2 \sim {\textrm{Beta}}\left( \frac{\phi }{2},\frac{\phi k}{2} \right) , \end{aligned}$$

where $\phi =1$ for ${\mathbb {F}} = \mathbb R$ and $\phi =2$ for ${\mathbb {F}}=\mathbb C$.

#### Proof

We will only prove the distribution for $\alpha$; the derivation for $\beta$ is entirely analogous. By the unitary invariance of the uniform distribution over the Stiefel manifold we may assume without loss of generality that $X_1 = [e_1\ \ldots \ e_k]$ and $x = e_{k+1}$, where $e_i$ is the *i*-th vector of the standard canonical basis. By Theorem 7, the matrix *V* is obtained from the QR factorization $\varOmega = VR$ of an $n \times k$ Gaussian random matrix $\varOmega$, with *R* being invertible almost surely. We partition

$$\begin{aligned} V^* = \begin{bmatrix} V_1&v_2&\cdots \end{bmatrix} = R^{-*} \begin{bmatrix} \varOmega _1&\omega _2&\cdots \end{bmatrix} = R^{-*} \varOmega ^*, \end{aligned}$$

such that $V_1,\varOmega _1$ are $k\times k$ matrices and $v_2,\omega _2$ are vectors. Then

$$\begin{aligned} 0 = V^* x_i = V^*X_1 a + V^*x \alpha = \begin{bmatrix} V_1&v_2 \end{bmatrix} \begin{bmatrix} a \\ \alpha \end{bmatrix} = R^{-*} \begin{bmatrix} \varOmega _1&\omega _2 \end{bmatrix} \begin{bmatrix} a \\ \alpha \end{bmatrix}. \end{aligned}$$

Since submatrices of Gaussian random matrices are again Gaussian random matrices, this means that $$\begin{bmatrix} a \\ \alpha \end{bmatrix}$$ is in the nullspace of a $k \times (k+1)$ Gaussian random matrix and has norm 1. Thus, the result on the distribution of $\alpha$ follows from Lemma 2.

The independence of $|\alpha |$ and $|\beta |$ follows from the independence of *U* and *V* combined with the fact that $|\alpha |$ does not depend on *U* and $|\beta |$ does not depend on *V*. $\square$

#### Remark 8

It is important to emphasize that the case ${\mathbb {F}}=\mathbb R$ in Proposition 3 not only requires $A,B,D_A,D_B$ to be real but also the eigenvalue $\lambda _i$ to be real.

#### Remark 9

An analysis similar to the one above was performed in [18, Proposition 6.5] and [17, Section 4.1] for unstructured perturbations. This analysis also starts from the relation (14) and then analyzes the distribution of $|\alpha |\cdot |\beta |$. One significant difference in our case is that $\alpha$ and $\beta$ are independent due to the structure of the perturbation in (10), while this does not hold for the setting considered in [17, 18].

### Statistics of $|\alpha | \cdot |\beta |$

As explained above, we aim at showing that the random variable $|\alpha ||\beta |$ is unlikely to become tiny. We start by computing the expected value of $|\alpha ||\beta |$. Since $|\alpha |$ and $|\beta |$ are independent random variables, we have ${\mathbb {E}}[|\alpha ||\beta |]={\mathbb {E}}[|\alpha |]{\mathbb {E}}[|\beta |]$. The factors can be computed using the following result from [18, Lemma A.1].

#### Lemma 3

Let $X\sim {\textrm{Beta}}(a,b)$, where $a,b>0$. Then

$$\begin{aligned} {\mathbb {E}}\big [X^{1/2}\big ]=\frac{\textrm{B}(a+1/2,b)}{\textrm{B}(a,b)},\quad \text {where}\quad \textrm{B}(a,b)=\frac{\varGamma (a)\varGamma (b)}{\varGamma (a+b)}. \end{aligned}$$

To simplify the presentation, we will from now on denote the scalars $\alpha$ and $\beta$, in the setting of Proposition 3, as $\alpha _{{\mathbb {F}}}$ and $\beta _{{\mathbb {F}}}$ with ${\mathbb {F}} \in \{\mathbb R, \mathbb C\}$. Combining Proposition 3 and Lemma 3 gives the following result.

#### Lemma 4

Under the assumptions of Proposition 3, the following holds:

- $$\displaystyle {\mathbb {E}}[|\alpha _\mathbb C|]={\mathbb {E}}[|\beta _\mathbb C|]=\frac{\sqrt{\pi }\varGamma (k+1)}{2\varGamma (k+3/2)}$$ and $$\displaystyle {\mathbb {E}}\left[ |\alpha _\mathbb C||\beta _\mathbb C|\right] =\frac{\pi \varGamma (k+1)^2}{4 \varGamma (k+3/2)^2}$$.
- $$\displaystyle {\mathbb {E}}[|\alpha _\mathbb R|]={\mathbb {E}}[|\beta _\mathbb R|]=\frac{\varGamma ((k+1)/2)}{\sqrt{\pi }\varGamma ((k+2)/2)}$$ and $$\displaystyle {\mathbb {E}}\left[ |\alpha _\mathbb R||\beta _\mathbb R|\right] =\frac{\varGamma ((k+1)/2)^2}{\pi \varGamma ((k+2)/2)^2}$$.

Table 1 contains the computed expected values for different *k*, using the results of Lemma 4.

Using the well-known bounds

16 $$\begin{aligned} \sqrt{x}\le \frac{\varGamma (x+1)}{\varGamma (x+1/2)}\le \sqrt{x+1/2} \end{aligned}$$

for $x>0$, the expected values of $|\alpha ||\beta |$ from Lemma 4 can be bounded as

17 $$\begin{aligned} \frac{\pi }{4(k+1)}\le {\mathbb {E}}[|\alpha _\mathbb C||\beta _\mathbb C|] \le \frac{\pi }{4(k+1/2)}, \quad \frac{2}{\pi (k+1)}\le {\mathbb {E}}[|\alpha _\mathbb R||\beta _\mathbb R|] \le \frac{2}{\pi k}. \end{aligned}$$

**Table 1:** Expected values of $|\alpha ||\beta |$ for $k=n-\textrm{nrank}(A,B)=1,2,4,\ldots ,64$

*k*	${\mathbb {E}}\left[ |\alpha _\mathbb C||\beta _\mathbb C|\right]$	${\mathbb {E}}\left[ |\alpha _\mathbb R||\beta _\mathbb R|\right]$
1	0.44444	0.40528
2	0.28444	0.25000
4	0.16512	0.14063
8	0.08972	0.07477
16	0.04688	0.03857
32	0.02398	0.01959
64	0.01213	0.00987

One therefore expects that $\gamma _i$ underestimates the true values $\gamma (\lambda _i)$ by roughly a factor 1/*k*.

For real matrices *A* and *B*, one would prefer to use real matrices in the perturbation (10) as well, because eigenvalue computations are performed more efficiently in real arithmetic. As we can see from Table 1 as well as from the bounds (17), the expected value of $|\alpha ||\beta |$ for real perturbations is only slightly smaller than the one for complex perturbations. However, as we will see in the following, the left tail of $|\alpha ||\beta |$ is less favorable in the real case and it appears to be safer to use complex modifications of the original pencil even for real data.

### Bounds on left tail of $|\alpha | \cdot |\beta |$

We will start with a simple tail bound that extends a result from [17, Proposition 4.3] to the complex case.

#### Corollary 3

Under the assumptions of Proposition 3, we have

18 $$\begin{aligned} {\mathbb {P}}(|\alpha _\mathbb C| |\beta _\mathbb C| < t) \le 2kt \end{aligned}$$

and

19 $$\begin{aligned} {\mathbb {P}}(|\alpha _\mathbb R| |\beta _\mathbb R| < t) \le \sqrt{8k t/\pi } \end{aligned}$$

for every $$0\le t\le 1$$.

#### Proof

Using $\min \{|\alpha |^4,|\beta |^4\} \le |\alpha |^2 |\beta |^2$, we obtain

$$\begin{aligned} {\mathbb {P}}\left( |\alpha |^2 |\beta |^2< t^2\right)&\le {\mathbb {P}}\left( \min \left\{ |\alpha |^4,|\beta |^4\right\}< t^2\right) \le {\mathbb {P}}\left( |\alpha |^2< t\right) + {\mathbb {P}}\left( |\beta |^2 < t\right) \\&= \frac{2}{{\textrm{B}}(\phi /2,\phi k/2)} \int _0^t x^{\phi /2-1} (1-x)^{\phi k/2-1}\, \textrm{d}x, \end{aligned}$$

where $\phi =1$ for ${\mathbb {F}} = \mathbb R$ and $\phi =2$ for ${\mathbb {F}}=\mathbb C$. For $\phi = 2$, we obtain from $|1-x|\le 1$ that

$$\begin{aligned} {\mathbb {P}}\left( |\alpha _\mathbb C|^2 |\beta _\mathbb C|^2 < t^2\right) \le \frac{2t}{{\textrm{B}}(1, k)} = 2k t. \end{aligned}$$

Similarly, for $\phi = 1$ we get

$$\begin{aligned} {\mathbb {P}}\left( |\alpha _\mathbb R|^2 |\beta _\mathbb R|^2 < t^2\right) \le \frac{4\sqrt{t}}{{\textrm{B}}(1/2, k/2)} \le \sqrt{\frac{8k t}{\pi }}, \end{aligned}$$

where we used the bound (16) to derive the last inequality. $\square$

Although we know from Proposition 3 that $|\alpha |$ and $|\beta |$ are independent random variables, this is not used in Corollary 3. The following proposition, where we exploit the fact that $|\alpha |$ and $|\beta |$ are independent, significantly improves the results of Corollary 3.

#### Proposition 4

Under the assumptions of Proposition 3, it holds that

20 $$\begin{aligned} {\mathbb {P}}\left( |\alpha _\mathbb C| |\beta _\mathbb C| < t\right) \le k^2t^2(1-2\ln t) \end{aligned}$$

and

21 $$\begin{aligned} {\mathbb {P}}\left( |\alpha _\mathbb R| |\beta _\mathbb R| < t\right) \le \frac{2k}{\pi } t(-\ln t)+{{\mathcal {O}}}(t), \quad t \rightarrow 0. \end{aligned}$$

#### Proof

To derive the bound (20) we first observe that, since $|\alpha _\mathbb C|$ and $|\beta _\mathbb C|$ are independent by Proposition 3, it holds that

$$\begin{aligned} {\mathbb {P}}(|\alpha _\mathbb C| |\beta _\mathbb C|\le t)= {\mathbb {P}}\left( |\alpha _\mathbb C|^2 |\beta _\mathbb C|^2\le t^2\right) = \iint _{{\mathcal {D}}}g(x;k)g(y;k)\,\textrm{d}x\,\textrm{d}y \end{aligned}$$

with ${{\mathcal {D}}}=\left\{ (x,y)\in [0,1]\times [0,1]:\ xy\le t^2\right\}$ and $g(x;k)=\frac{1}{\textrm{B}(1,k)}(1-x)^{k-1}.$ Using the bound $g(x;k)\le \frac{1}{\textrm{B}(1,k)}=k$ gives (20):

$$\begin{aligned} {\mathbb {P}}(|\alpha _\mathbb C| |\beta _\mathbb C|\le t) \le k^2 \iint _{{\mathcal {D}}}\textrm{d}x\textrm{d}y = k^2 t^2(1-2\ln {t}). \end{aligned}$$

For real perturbations we have $|\alpha _\mathbb R|^2, |\beta _\mathbb R|^2\sim {\textrm{Beta}}(1/2,k/2)$ with the distribution function satisfying

$$\begin{aligned} h(x;k)=\frac{1}{\textrm{B}(1/2,k/2)}x^{-1/2}(1-x)^{k/2-1} \le \frac{1}{\textrm{B}(1/2,k/2)}x^{-1/2}. \end{aligned}$$

This gives

$$\begin{aligned} {\mathbb {P}}(|\alpha _\mathbb R| |\beta _\mathbb R|\le t)&= \iint _{{\mathcal {D}}}h(x;k)h(y;k)\textrm{d}x\textrm{d}y\\&\le \frac{1}{B(1/2,k/2)^2} \iint _{{\mathcal {D}}}x^{-1/2}y^{-1/2}\textrm{d}x\textrm{d}y\\&=\frac{1}{B(1/2,k/2)^2} 4 t\left( 2 \sqrt{\pi } - 1 + t -\ln {t}\right) \\&\le \frac{2 k}{\pi } t\left( 2 \sqrt{\pi } - 1 + t -\ln {t}\right) , \end{aligned}$$

where we applied (16). $\square$

Proposition 4 indicates that, even for a real singular pencil, it would be better to use complex perturbations as they give a much smaller probability of obtaining tiny $|\alpha ||\beta |$. This is confirmed by the following lower bound for ${\mathbb {P}}(|\alpha _\mathbb R| |\beta _\mathbb R|< t)$.

#### Lemma 5

Under the assumptions of Proposition 3, it holds that

22 $$\begin{aligned} {\mathbb {P}}\left( |\alpha _\mathbb R| |\beta _\mathbb R| < t\right) \ge \sqrt{\frac{8(k-1)}{\pi }} t+{{\mathcal {O}} }(t^2), \quad t\rightarrow 0. \end{aligned}$$

#### Proof

It is easy to see that

$$\begin{aligned} {\mathbb {P}}(|\alpha _\mathbb R| |\beta _\mathbb R|< t)&\ge {\mathbb {P}}(|\alpha _\mathbb R|<t) + \mathbb P(|\beta _\mathbb R|<t) -\mathbb P(|\alpha _\mathbb R|<t)\mathbb P(|\beta _\mathbb R|<t)\\&= 2\, \mathbb P(|\alpha _\mathbb R|<t)-{\mathbb {P}}(|\alpha _\mathbb R|<t)^2. \end{aligned}$$

From the Taylor expansion of $\mathbb P(|\alpha _\mathbb R|<t)$ around $t=0$ we get

$$\begin{aligned} {\mathbb {P}}(|\alpha _\mathbb R| |\beta _\mathbb R| < t)\ge \frac{4}{\textrm{B}(1/2,k/2)}t +{{\mathcal {O}}}(t^2) \end{aligned}$$

and the bound follows from applying (16). $\square$

From Lemma 5 and Proposition 4 we see that for sufficiently small $t>0$ the lower bound (22) for ${\mathbb {P}}\left( |\alpha _\mathbb R| |\beta _\mathbb R| < t\right)$ is much larger than the upper bound (20) for ${\mathbb {P}}\left( |\alpha _\mathbb C| |\beta _\mathbb C| < t\right)$.

**Fig. 1:**
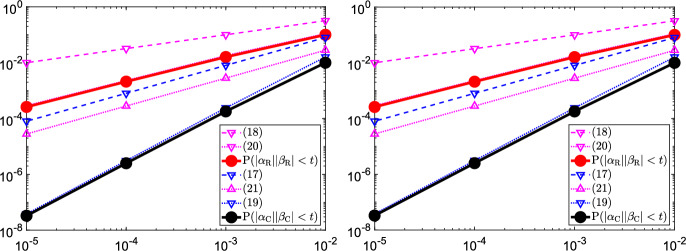
Comparison of bounds and actual values of ${\mathbb {P}}(|\alpha ||\beta |<t)$ for $t=10^{-5},10^{-4},10^{-3},10^{-2}$, and for $k=4$ (left) and $k=8$ (right)

Figure 1 compares the obtained bounds to ${\mathbb {P}}\left( |\alpha _\mathbb C| |\beta _\mathbb C| < t\right)$ and ${\mathbb {P}}\left( |\alpha _\mathbb R| |\beta _\mathbb R| < t\right)$, computed for $k=4$ and $k=8$ using the probability density functions from Appendix A. The solid black line corresponds to ${\mathbb {P}}\left( |\alpha _\mathbb C| |\beta _\mathbb C| < t\right)$. The blue dotted and dashed lines are the refined bound (20) from Proposition 4 and the simple upper bound (18) from Corollary 3, respectively. The solid red line corresponds to ${\mathbb {P}}\left( |\alpha _\mathbb R| |\beta _\mathbb R| < t\right)$. The corresponding bounds are magenta curves, which show the lower bound (22) from Lemma 5, the refined upper bound (21) from Proposition 4, and the simple upper bound (19) from Corollary 3, respectively, As expected, the bounds from Proposition 4 are much sharper than the simple bounds from Corollary 3. Also, it is clearly seen from Fig. 1 that the probability of obtaining a tiny value for $|\alpha ||\beta |$ is much larger when using real perturbations.

*Summary* Together with the discussion in the beginning of this section, Proposition 4 allows us to compare the (reciprocal) eigenvalue sensitivity $\gamma (\lambda _i)$ of the original pencil with the corresponding quantity $\gamma _i$ for any of the three modified pencils used in Algorithms 1–3. For complex perturbations, we obtain that

$$\begin{aligned} \gamma _i \le \gamma (\lambda _i) \le \gamma _i / t \end{aligned}$$

holds with probability at least $1-k^2t^2(1-2\ln t)$ for any $t > 0$.

## Numerical examples

All numerical examples were obtained with Matlab 2021b [19]. We used the implementations of Algorithms 1–3 available as routine singgep in MultiParEig [25].

### Example 1

We consider the $8\times 8$ singular matrix pencil

23 $$\begin{aligned} {\small A={\left[ \begin{array}{rrrrrrrr} -1 &{} -1 &{} -1 &{} -1 &{} -1 &{} -1 &{} -1 &{} 0\\ 1 &{} 0 &{} 0 &{} 0 &{} 0 &{} 0 &{} 0 &{} 0\\ 1 &{} 2 &{} 1 &{} 1 &{} 1 &{} 1 &{} 1 &{} 0\\ 1 &{} 2 &{} 3 &{} 3 &{} 3 &{} 3 &{} 3 &{} 0\\ 1 &{} 2 &{} 3 &{} 2 &{} 2 &{} 2 &{} 2 &{} 0\\ 1 &{} 2 &{} 3 &{} 4 &{} 3 &{} 3 &{} 3 &{} -1\\ 1 &{} 2 &{} 3 &{} 4 &{} 5 &{} 5 &{} 4 &{} 1\\ 0 &{} 0 &{} 0 &{} 0 &{} 2 &{} 2 &{} 1 &{} 2\end{array}\right] },\quad B={\left[ \begin{array}{rrrrrrrr} -2 &{} -2 &{} -2 &{} -2 &{} -2 &{} -2 &{} -2 &{} 0 \\ 2 &{} -1 &{} -1 &{} -1 &{} -1 &{} -1 &{} -1 &{} 0 \\ 2 &{} 5 &{} 5 &{} 5 &{} 5 &{} 5 &{} 5 &{} 0\\ 2 &{} 5 &{} 5 &{} 4 &{} 4 &{} 4 &{} 4 &{} 0\\ 2 &{} 5 &{} 5 &{} 6 &{} 5 &{} 5 &{} 5 &{} -1\\ 2 &{} 5 &{} 5 &{} 6 &{} 7 &{} 7 &{} 7 &{} 1\\ 2 &{} 5 &{} 5 &{} 6 &{} 7 &{} 6 &{} 6 &{} 1\\ 0 &{} 0 &{} 0 &{} 0 &{} 0 &{} -1 &{} -1 &{} 0\end{array}\right] }},\nonumber \\ \end{aligned}$$

which is constructed so that the KCF contains blocks of all four possible types. It holds that $\textrm{nrank}(A,B)=6$, and the KCF has blocks $J_1(1/2)$, $J_1(1/3)$, $N_1$, $L_0$, $L_1$, $L_0^T$, and $L_2^T$.

**Fig. 2:**
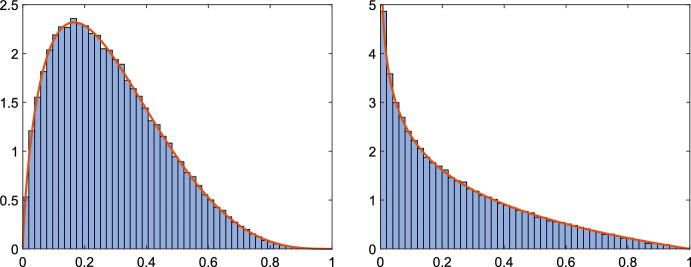
Example 1: Histogram of $\gamma _1/\gamma (\lambda _1)$ and pdf using complex (left) and real (right) modifications

Algorithm 1 was applied $10^5$ times using random real and complex modifications; we compared the computed values of $\gamma _1$ to the exact value of $\gamma (\lambda _1)$ for the eigenvalue $\lambda _1=1/3$. The histograms of $\gamma _1/\gamma (\lambda _1)$ for real and complex modifications together with the corresponding probability density function (pdf) from Appendix A are presented in Fig. 2. The histograms appear to be consistent with the pdfs. The computed average values of $|\alpha ||\beta |$ are 0.28437 for complex and 0.24934 for real modifications, which are both close to the theoretically predicted values for $k=2$ in Table 1. We note that we get almost identical results for the other eigenvalue $\lambda _2=1/2$.

### Example 2

For the second example we consider the pencil $\varDelta _1-\lambda \varDelta _0$ from [12, Ex. 7.1] with matrices

$$\begin{aligned}\varDelta _0=B_1\otimes C_2-C_1\otimes B_2,\quad \varDelta _1=C_1\otimes A_2-A_1\otimes C_2 \end{aligned}$$

of size $25\times 25$ related to the two-parameter eigenvalue problem (for details see, e.g., [12, Sec. 7]) of the form

$$\begin{aligned} A_1+\lambda B_1+\mu C_1&=\small {\left[ \begin{array}{ccccc} 0 &{} 0 &{} 4 + 7\lambda &{} 1 &{} 0\\ 0 &{} 5 + 8\lambda &{} 2 &{} -\lambda &{} 1 \\ 6 + 9\lambda + 10\mu &{} 3 &{} 1 &{} 0 &{} -\lambda \\ 1 &{} -\mu &{} 0 &{} 0 &{} 0\\ 0 &{} 1 &{} -\mu &{} 0 &{} 0 \end{array}\right] }, \\ A_2+\lambda B_2+\mu C_2&=\small {\left[ \begin{array}{ccccc} 0 &{} 0 &{} 7 + 4\lambda &{} 1 &{} 0\\ 0 &{} 6 + 3\lambda &{} 9 &{} -\lambda &{} 1 \\ 5 + 2\lambda + \mu &{} 8 &{} 10 &{} 0 &{} -\lambda \\ 1 &{} -\mu &{} 0 &{} 0 &{} 0\\ 0 &{} 1 &{} -\mu &{} 0 &{} 0 \end{array}\right] ,} \end{aligned}$$

where

$$\begin{aligned} \det (A_1+\lambda B_1+\mu C_1)&= 1 + 2\lambda + 3\mu + 4\lambda ^2 + 5\lambda \mu + 6\mu ^2 + 7\lambda ^3 + 8\lambda ^2\mu + 9\lambda \mu ^2 + 10\mu ^3,\\ \det (A_2+\lambda B_2+\mu C_2)&= 10 + 9\lambda + 8\mu + 7\lambda ^2 + 6\lambda \mu + 5\mu ^2 + 4\lambda ^3 + 3\lambda ^2\mu + 2\lambda \mu ^2 + \mu ^3. \end{aligned}$$

The normal rank of $\varDelta _1-\lambda \varDelta _0$ is 21 and the KCF contains 4 $L_0$, 4 $L_0^T$, 2 $N_4$, 1 $N_2$, 2 $N_1$, and 9 $J_1$ blocks. Its finite eigenvalues are $\lambda$-components of the 9 solutions of the system of two bivariate polynomials $\det (A_1+\lambda B_1+\mu C_1)=0$ and $\det (A_2+\lambda B_2+\mu C_2)=0$. The pencil $\varDelta _1-\lambda \varDelta _0$ has one real eigenvalue $\lambda _1=-2.41828$ and eight complex eigenvalues $\lambda _2,\ldots ,\lambda _9$.

**Fig. 3:**
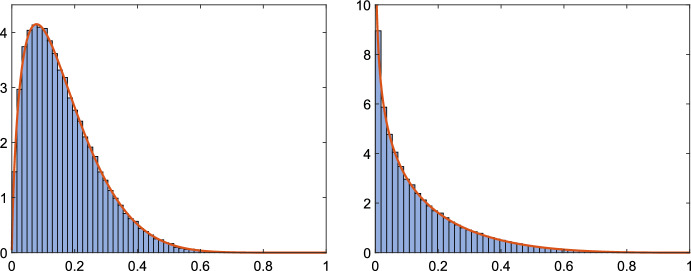
Example 2: Histogram of $\gamma _1/\gamma (\lambda _1)$ and pdf using complex (left) and real (right) projections

Algorithm 2 was applied $10^5$ times using random real and complex projections. We compared the computed values of $\gamma _1$ to the exact value of $\gamma (\lambda _1)$ for the real eigenvalue $\lambda _1$. In both cases the method successfully computed all finite eigenvalues. The histograms of $\gamma _1/\gamma (\lambda _1)$ together with the corresponding pdf from Appendix A are presented in Fig. 3. Similar to the previous example, both histograms appear to be consistent with the pdfs. The computed average values of $|\alpha ||\beta |$ are 0.16413 for complex and 0.14124 for real projections; both are again very close to the values in Table 1 for $k=4$.

Let us note that we get essentially identical results if we exchange the methods and use Algorithms 2 or 3 in Example 1 and Algorithms 1 or 3 in Example 2, as expected by Corollary 1. However, if we apply Algorithm 1 or 3 with real modifications or Algorithm 2 with real projections to the real pencil $\varDelta _1-\lambda \varDelta _0$, and consider any of the 8 complex eigenvalues $\lambda _2,\ldots ,\lambda _9$, we get results that cannot be explained by Proposition 3 and behave like the results in the next example.

### Example 3

In this example we study the effect of real projections on a complex eigenvalue of a real pencil, which is a situation that is not covered by Proposition 3. By considering two equivalent singular pencils we will show that the distribution function for $|\alpha _\mathbb R||\beta _\mathbb R|$, when we apply real projections to complex eigenvalues of real pencils, depends on more than just the difference *k* between the size of the pencil and its normal rank, which is the key value in Proposition 3.

We take block diagonal matrices

24 $$\begin{aligned} A_0={\left[ \begin{array}{rr} e_1e_5^T &{} \\ &{} A_{20} \end{array}\right] },\quad B_0={\left[ \begin{array}{rr} e_1e_4^T &{} \\ &{} B_{20} \end{array}\right] }, \end{aligned}$$

where $e_1,e_4,e_5$ are standard basis vectors in $\mathbb R^5$, and

$$\begin{aligned}A_{20}=\left[ \begin{array}{rrrrr} 1 &{} 0 &{} 0 &{} 0 &{} 0 \\ 0 &{} 2 &{} 0 &{} 0 &{} 0 \\ 0 &{} 0 &{} 1 &{} 1 &{} 0 \\ 0 &{} 0 &{} -1 &{} 1 &{} 0 \\ 0 &{} 0 &{} 0 &{} 0 &{} 1 \end{array}\right] ,\quad B_{20}=\left[ \begin{array}{rrrrr} 1 &{} 0 &{} 0 &{} 0 &{} 0 \\ 0 &{} 1 &{} 0 &{} 0 &{} 0 \\ 0 &{} 0 &{} 1 &{} 0 &{} 0 \\ 0 &{} 0 &{} 0 &{} 1 &{} 0 \\ 0 &{} 0 &{} 0 &{} 0 &{} 0 \end{array}\right] . \end{aligned}$$

The $10\times 10$ pencil $A_0-\lambda B_0$ has $\textrm{nrank}(A_0,B_0)=6$ and the KCF has blocks $J_1(1+\textrm{i})$, $J_1(1-\textrm{i})$, $J_1(2)$, $N_1$, 3 $L_0$, $L_1$, 3 $L_0^T$, and $L_1^T$. We multiply $A_0$ and $B_0$ into $A_i=Q_iA_0Z_i$ and $B_i=Q_iB_0Z_i$ by real matrices $Q_i$ and $Z_i$ whose entries are independent random variables uniformly distributed on (0, 1) for $i=1,2$ to get two equivalent singular pencils of the same size, normal rank and eigenvalues.

**Fig. 4:**
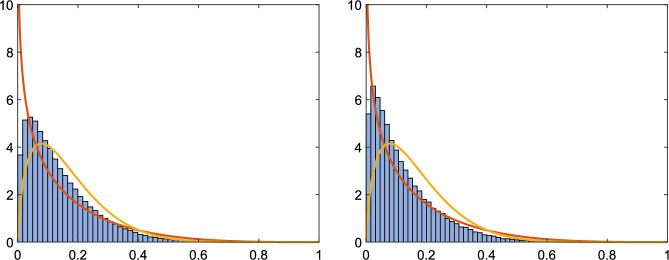
Example 3: Histogram of $\gamma _1/\gamma (\lambda _1)$ and theoretical distribution functions (real and complex) for $k=4$ using real perturbations for a complex eigenvalue of a real pencil for $A_1-\lambda B_1$ (left) and $A_2-\lambda B_2$ (right)

Algorithm 2 was applied $10^5$ times using random real projections to each of the pencils $A_1-\lambda B_1$ and $A_2-\lambda B_2$ and the computed value of $\gamma _1$ was compared to the exact value of $\gamma (\lambda _1)$ for the complex eigenvalue $\lambda _1=1+\textrm{i}$. The histograms of $\gamma _1/\gamma (\lambda _1)$ together with the theoretical distribution functions from Sect. A are presented in Figure 4. We see that, although the pencils $A_1-\lambda B_1$ and $A_2-\lambda B_2$ are equivalent, the histograms are different. The histograms also look different than in the case when we use real projections for a real eigenvalue for $k=4$ (Fig. 2 right) or complex projections (Fig. 2 left). While the shape of the left histogram in Fig. 4 resembles the shape expected for complex perturbations, is the shape of the right histogram more in line with the distribution function for real perturbations. The computed average values of $|\alpha _\mathbb R||\beta _\mathbb R|$ are also different, we get 0.12195 for $A_1-\lambda B_1$ and 0.13623 for $A_2-\lambda B_2$, both values are completely different from the values in Table 1 for $k=4$.

Since we get histograms of different shape for two real equivalent pencils, this shows that the distribution function for $|\alpha _\mathbb R||\beta _\mathbb R|$, when we apply real perturbations to complex eigenvalues of real pencils, depends on more than just the structure of the KCF.

We remark that the histograms (not shown) for the real eigenvalue $\lambda _3=2$ for both pencils look identical to the right picture in Fig. 3 from Example 2, where $k=4$ as well, which agrees with Remark 3 that even if some eigenvalues of the real pencil are complex, this does not affect the behavior of real perturbations to real eigenvalues. The computed average values of $|\alpha _\mathbb R||\beta _\mathbb R|$ for the real eigenvalue $\lambda _3$ are 0.14077 for $A_1-\lambda B_1$ and 0.14110 for $A_2-\lambda B_2$, they both agree with the value in Table 1 for $k=4$.

The last numerical example reflects that the case of real perturbations for a complex eigenvalue of a real singular pencil is not covered by Proposition 3. Indeed, this case was not taken properly into account in [18] and it remains an open problem to derive an expression or a tight simple bound for the $\delta$-weak condition number of a complex eigenvalue under real perturbations.

## Conclusions

We have analyzed three random based numerical methods for computing finite eigenvalues of a singular matrix pencil. All algorithms are based on random matrices that transform the original singular pencil into a regular one in such way that the eigenvalues remain intact. Our analysis confirms the numerical validity of these methods with high probability.

We also obtained sharp left tail bounds on the distribution of a product of two independent random variables distributed with the generalized beta distribution of the first kind or Kumaraswamy distribution.

## Figures and Tables

**Fig. 5:**
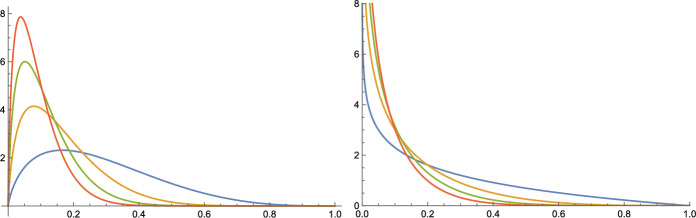
Pdfs $f_\mathbb C(x;k)$ (left) and $f_\mathbb R(x;k)$ (right) for $k=2,4,6,8$

## A closer look at the distribution of $|\alpha ||\beta |$

In this section, we derive explicit expressions for the probability density function (pdf) of $|\alpha ||\beta |$. For this purpose, we first write down the pdfs of $|\alpha |$, $|\beta |$, which we can express with the generalized beta distribution of the first kind [20] in the real case and the Kumaraswamy distribution [14] in the complex case.

### Lemma 6

Under the assumptions of Proposition 3, we have

- $|\alpha _\mathbb C|, |\beta _\mathbb C|\sim {\textrm{GB}}_1(2,1,1,k)={\textrm{Kumaraswamy}}(2,k)$ with the pdf
  $$\begin{aligned} h(x;k)=2k x(1-x^2)^{k-1}, \end{aligned}$$
- $|\alpha _\mathbb R|, |\beta _\mathbb R| \sim {\textrm{GB}}_1(2,1,\frac{1}{2},\frac{k}{2})$ with the pdf
  $$\begin{aligned} g(x;k)=\frac{2}{B(\frac{1}{2},\frac{k}{2})}(1-x^2)^{k/2-1}, \end{aligned}$$

where ${\textrm{GB}}_1(a,b,p,q)$ is the generalized beta distribution of the first kind and $\textrm{Kumaraswamy}(p,q)$ denotes the Kumaraswamy distribution with parameters *p* and *q*.

### Proof

The pdf of $X\sim {\textrm{Beta}}(p,q)$ is given by

$$\begin{aligned}f(x;p,q)=\frac{1}{\textrm{B}(p,q)}x^{p-1}(1-x)^{q-1}.\end{aligned}$$

It follows that the pdf for $Y = X^{1/2}$ is

$$\begin{aligned}2yf(y^2;p,q)=\frac{2}{\textrm{B}(p,q)}y^{2p-1}(1-y^2)^{q-1}.\end{aligned}$$

Now, we apply Proposition 3 and insert the corresponding values of *p*, *q*. The proof is concluded by identifying the obtained densities with the Kumaraswamy distribution in the complex case and the generalized beta distribution of the first kind in the real case; see, e.g., [14, 20]. $\square$

The refs [14, 20] used in the proof above also provide expressions for the moments of the two distributions in Lemma 6, which can be used to verify the results of Lemma 4.

In order to derive the pdf of $|\alpha |\cdot |\beta |$ from the result of Lemma 6, we have performed symbolic computations in Wolfram Mathematica [31]. Let $f_\mathbb C(x;k)$ and $f_\mathbb R(x;k)$ denote the pdfs for $|\alpha _\mathbb C| |\beta _\mathbb C|$ and $|\alpha _\mathbb R| |\beta _\mathbb R|$, respectively. Using that $|\alpha _\mathbb C|, |\beta _\mathbb C|$ are independent and $|\alpha _\mathbb C|, |\beta _\mathbb C|\sim {\textrm{Kumaraswamy}}(2,k)$, we obtain that

$$\begin{aligned} f_\mathbb C(x;k)&= 2k^2 x(x^2-1)^{2k-1}\textrm{B}(k,k+1)\Big (\frac{k(x^2-1)}{2k+2} {}_{2}F_1(k+1,k+1,2k+2,1-x^2)\\&\quad -2{}_2F_1(k,k,2k+1,1-x^2)\Big ), \end{aligned}$$

where ${}_{2}F_1(a,b,c,z)$ is the hypergeometric function. One finds that $f_\mathbb C$ takes the form

25 $$\begin{aligned} f_\mathbb C(x;k) = x\big ( p_{k-1}(x^2)+ q_{k-1}(x^2)\ln x\big ), \end{aligned}$$

where $p_{k-1}$ and $q_{k-1}$ are polynomials of degree $k-1$. Some explicit expressions for small *k* are

$$\begin{aligned} f_\mathbb C(x;1)&= -4x \ln x,\\ f_\mathbb C(x;2)&= 16x \big (-1+x^2 - (1+x^2)\ln x\big ),\\ f_\mathbb C(x;3)&= 18x \big (-3+3x^4 - 2(1+4x^2+x^4)\ln x\big ),\\ f_\mathbb C(x;4)&= \frac{32}{3} x\big (-11-27x^2+27x^4+11x^6-6(1+9x^2+9x^4+x^6)\ln x\big ).\\ \end{aligned}$$

We were unable to obtain a closed form for the distribution of $|\alpha _\mathbb R| |\beta _\mathbb R|$, with $|\alpha _\mathbb R|, |\beta _\mathbb R|$ independent and $|\alpha _\mathbb R|, |\beta _\mathbb R| \sim {\textrm{GB}}_1(2,1,\frac{1}{2},\frac{k}{2})$. For $k=2\,m$ we conjecture that

$$\begin{aligned} f_\mathbb R(x;2m) = p_{m-1}(x^2)+ q_{m-1}(x^2)\ln x, \end{aligned}$$

where $p_{m-1}$ and $q_{m-1}$ are polynomials of degree $m-1$. In addition to the fact that this looks similar to (25), this conjecture is also supported by the following expressions for small *m*:

$$\begin{aligned} f_\mathbb R(x;2)&= -\ln x,\\ f_\mathbb R(x;4)&= \frac{9}{4}\big (-1+x^2-(1+x^2)\ln x\big ),\\ f_\mathbb R(x;6)&= \frac{225}{128} \big (-3+3x^4-2(1+4x^2+x^4)\ln x\big ). \end{aligned}$$

The graphs of $f_\mathbb C(x;k)$ and $f_\mathbb R(x;k)$ for $k=2,4,6,8$ are presented in Fig. 5. Note that $\lim _{x\rightarrow 0}f_\mathbb C(x;k)=0$ and $\lim _{x\rightarrow 0}f_\mathbb R(x;k)=\infty$.

## Footnotes

1 Although this heuristics works very well in practice, an analysis is beyond the scope of this work. Already the seemingly simpler special case of numerically deciding whether $A-\lambda B$ is close to a singular pencil is quite intricate [16].

## References

[1] De Terán, F., Dopico, F.M., Moro, J.: First order spectral perturbation theory of square singular matrix pencils. Linear Algebra Appl. **429**(2–3), 548–576 (2008)

[2] Demmel, J., Kågström, B.: The generalized Schur decomposition of an arbitrary pencil : robust software with error bounds and applications. I. Theory and algorithms. ACM Trans. Math. Softw. **19**(2), 160–174 (1993)

[3] Demmel, J., Kågström, B.: The generalized Schur decomposition of an arbitrary pencil : robust software with error bounds and applications. II. Software and applications. ACM Trans. Math. Softw. **19**(2), 175–201 (1993)

[4] Dixon, J.D.: Estimating extremal eigenvalues and condition numbers of matrices. SIAM J. Numer. Anal. **20**(4), 812–814 (1983)

[5] Dopico, F.M., Noferini, V.: Root polynomials and their role in the theory of matrix polynomials. Linear Algebra Appl. **584**, 37–78 (2020)

[6] Eaton, M.L.: Multivariate Statistics. Wiley, New York (1983)

[7] Edelman, A., Ma, Y.: Staircase failures explained by orthogonal versal forms. SIAM J. Matrix Anal. Appl. **21**(3), 1004–1025 (2000)

[8] Frayssé, V., Toumazou, V.: A note on the normwise perturbation theory for the regular generalized eigenproblem. Numer. Linear Algebra Appl. **5**(1), 1–10 (1998)

[9] Gantmacher, F.R.: The Theory of Matrices. Vols. 1, 2. Chelsea Publishing Co., New York (1959). Translated by K. A. Hirsch

[10] Higham, D.J., Higham, N.J.: Structured backward error and condition of generalized eigenvalue problems. SIAM J. Matrix Anal. Appl. **20**(2), 493–512 (1999)

[11] Higham, N.J.: Accuracy and Stability of Numerical Algorithms, 2nd edn. SIAM, Philadelphia (2002)

[12] Hochstenbach, M.E., Mehl, C., Plestenjak, B.: Solving singular generalized eigenvalue problems by a rank-completing perturbation. SIAM J. Matrix Anal. Appl. **40**(3), 1022–1046 (2019)

[13] Hochstenbach, M.E., Mehl, C., Plestenjak, B.: Solving singular generalized eigenvalue problems. Part II: projection and augmentation. SIAM J. Matrix Anal. Appl. **44**(4), 1589–1618 (2023)

[14] Jones, M.C.: Kumaraswamy’s distribution: a beta-type distribution with some tractability advantages. Stat. Methodol. **6**(1), 70–81 (2009)

[15] Kågström, B., Kressner, D.: Multishift variants of the QZ algorithm with aggressive early deflation. SIAM J. Matrix Anal. Appl. **29**(1), 199–227 (2006)

[16] Kressner, D., Voigt, M.: Distance problems for linear dynamical systems. In: Numerical Algebra. Matrix Theory, Differential-Algebraic Equations and Control Theory, pp. 559–583. Springer, Cham (2015)

[17] Kressner, D., Šain Glibić, I.: Singular quadratic eigenvalue problems: linearization and weak condition numbers. BIT **63**(1), 18, 25 (2023)

[18] Lotz, M., Noferini, V.: Wilkinson’s bus: weak condition numbers, with an application to singular polynomial eigenproblems. Found. Comput. Math. **20**(6), 1439–1473 (2020)

[19] Matlab: The MathWorks Inc., Natick, MA, Version 2021b

[20] McDonald, J.B.: Some generalized functions for the size distribution of income. Econometrica **52**, 647–664 (1984)

[21] Mezzadri, F.: How to generate random matrices from the classical compact groups. Notices Am. Math. Soc. **54**(5), 592–604 (2007)

[22] Moler, C.B., Stewart, G.W.: An algorithm for generalized matrix eigenvalue problems. SIAM J. Numer. Anal. **10**, 241–256 (1973)

[23] Muhič, A., Plestenjak, B.: A method for computing all values such that has a multiple eigenvalue. Linear Algebra Appl. **440**, 345–359 (2014)

[24] Muirhead, R.J.: Aspects of Multivariate Statistical Theory. Wiley Series in Probability and Mathematical Statistics. Wiley, New York (1982)

[25] Plestenjak, B.: MultiParEig. Toolbox for multiparameter and singular eigenvalue problems. www.mathworks.com/matlabcentral/fileexchange/47844-multipareig

[26] Stewart, G.W.: The efficient generation of random orthogonal matrices with an application to condition estimators. SIAM J. Numer. Anal. **17**(3), 403–409 (1980). (**(loose microfiche suppl.)**)

[27] Stewart, G.W., Sun, J.-G.: Matrix Perturbation Theory. Academic Press, New York (1990)

[28] Van Dooren, P.: The computation of Kronecker’s canonical form of a singular pencil. Linear Algebra Appl. **27**, 103–140 (1979)

[29] Van Dooren, P.: Reducing subspaces: Definitions, properties and algorithms. In: Kågström, B., Ruhe, A. (eds.) Matrix Pencils, pp. 58–73. Springer, New York (1983)

[30] Wilkinson, J.H.: Kronecker’s canonical form and the algorithm. Linear Algebra Appl. **28**, 285–303 (1979)

[31] Wolfram Research Inc., Champaign, IL. Mathematica, Version 13.0

